# *Apolipoprotein E* ɛ3/ɛ4 genotype is associated with premature myocardial infarction: a hospital based retrospective study

**DOI:** 10.1186/s12872-026-05513-5

**Published:** 2026-01-15

**Authors:** Hao Wang, Bin Li, Wenhao Chen, Guoliang Wei, Kehui Chen, Weihong Wang, Yuanliang Liu

**Affiliations:** Center for Cardiovascular Diseases, Meizhou People’s Hospital, Meizhou Academy of Medical Sciences, Meizhou, China

**Keywords:** Premature myocardial infarction, APOE, Susceptibility, Risk

## Abstract

**Objective:**

To explore the association between *apolipoprotein E (APOE)* gene polymorphisms and the risk of premature (age of onset: men ≤ 55 years old, women ≤ 65 years old) myocardial infarction (PMI).

**Methods:**

This study retrospectively collected the medical records (age, gender, hypertension, diabetes mellitus, smoking, drinking, and serum lipid) of 379 PMI patients and 628 age-matched non-AMI individuals (controls), from December 2018 to March 2024. The relationship between *APOE* polymorphisms and PMI was analyzed.

**Results:**

15(1.5%) individuals carried ɛ2/ɛ2, 147(14.6%) had ɛ2/ɛ3, 16(1.6%) presented with ɛ2/ɛ4, 670(66.5%) were ɛ3/ɛ3 carriers, 149(14.8%) had ɛ3/ɛ4, and 10 (1.0%) carried ɛ4/ɛ4. The proportion of ɛ2/ɛ3 genotype was significantly lower in the PMI group than in controls (7.7% vs. 18.8%, *p* < 0.001), whereas the prevalence of ɛ3/ɛ4 genotype was substantially higher in the PMI group (20.6% vs. 11.3%, *p* < 0.001). Logistic regression analysis identified some associated factors: smoking (odds ratio [OR]: 3.057, 95% confidence interval [CI]: 2.098–4.455, *p* < 0.001), hypertension (OR: 4.474, 95% CI: 3.273–6.117, *p* < 0.001), and dyslipidemia (OR: 1.805, 95% CI: 1.333–2.443, *p* < 0.001). Additionally, genetic factors were associated with PMI: the *APOE* ɛ3/ɛ4 genotype (vs. ɛ3/ɛ3, OR: 1.548, 95% CI: 1.038–2.309, *p* = 0.032) and the presence of ɛ4 allele (vs. ɛ3, OR: 1.521, 95% CI: 1.033–2.241, *p* = 0.034) were confirmed as independent associated factors.

**Conclusions:**

*APOE* ε3/ε4 genotype was significantly associated with PMI, suggesting that this genotype could serve as a potential genetic marker for PMI risk assessment.

## Introduction

Myocardial Infarction (MI) is the acute and fatal manifestation of coronary atherosclerotic heart disease [1]. Premature Myocardial Infarction (PMI) is defined as MI that occurs in men ≤ 55 years old and women ≤ 65 years old [2–4]. PMI has a worse prognosis and significantly affects the health of the working population, making it a key concern in the field of cardiovascular medicine [5, 6]. Epidemiological data show that although the prevention and treatment methods for coronary heart disease have improved over the past decades, the incidence of MI still shows an upward trend in the young and middle-aged population [6]. The pathogenic mechanism of premature cardiovascular diseases involves complex interactions of genetic susceptibility, metabolic abnormalities, and environmental factors [7–9]. Among them, genetic factors play a particularly prominent role in PMI [10, 11].

Apolipoprotein E (ApoE) is a core protein that regulates lipid metabolism. It mediates the clearance of lipoprotein particles by binding to receptors such as low-density lipoprotein receptor (LDLR), directly influencing the homeostasis of key indicators such as plasma total cholesterol (TC) and low-density lipoprotein cholesterol (LDL-C) [12]. Beyond its canonical role in regulating lipid metabolism, ApoE also modulates the progression of atherosclerosis (AS) by participating in inflammatory responses [13, 14]. Emerging evidence indicated that ApoE can regulate the activation status of inflammatory cells such as macrophages and endothelial cells, thereby promoting the secretion of pro-inflammatory cytokines including tumor necrosis factor-α (TNF-α) and interleukin-6 (IL-6), which in turn amplifies local inflammatory reactions [15, 16]. Concurrently, vascular endothelial cells in individuals with ApoE dysfunction are prone to functional impairment, characterized by reduced nitric oxide (NO) synthesis and elevated endothelin-1 (ET-1) secretion [17, 18]. This endothelial dysfunction leads to diminished vasodilatory capacity and increased vascular permeability, further exacerbating inflammatory cell infiltration and plaque instability [19]. Additionally, ApoE influences the integrity of the atherosclerotic plaque fibrous cap via regulating the expression of matrix metalloproteinases (MMPs). Specifically, ApoE may modulate the activity of MMP-2 and MMP-9 to degrade collagen within the plaque fibrous cap, thereby increasing the risk of plaque rupture and ultimately triggering acute myocardial infarction [20, 21].

ApoE is encoded by the *APOE* gene. The *APOE* gene has significant polymorphism, consisting of six genotypes (ɛ2/ɛ2, ɛ2/ɛ3, ɛ2/ɛ4, ɛ3/ɛ3, ɛ3/ɛ4, and ɛ4/ɛ4) formed by the combination of three alleles: ε2, ε3, and ε4 [22–25]. The ε3/ε3 genotype and ε3 allele have the highest frequency in the population; the ε2 allele has a lower affinity for the receptor, resulting in delayed clearance of lipoproteins; while the ε4 allele is closely related to elevated LDL-C levels through altering the distribution and metabolic efficiency of lipoproteins [26]. A large number of studies have confirmed that the *APOE* polymorphisms are associated with cardiovascular diseases. The risk of myocardial infarction in individuals carrying the ε4 allele is higher than that in those carrying the ε3 allele, while the ε2 allele shows a protective effect [27, 28]. The mechanism may involve multiple pathways: ε4 genotype not only accelerates the formation of atherosclerotic plaques by increasing LDL-C [29], but also activates inflammatory signaling pathways and enhances oxidative stress responses, further promoting vascular wall damage and plaque instability [30].

Although the association between *APOE* polymorphisms and MI has been extensively studied, there are still gaps in the research on the relationship between PMI and specific genotypes. Firstly, most studies include cases of MI across all age groups and do not separately analyze the genotype effect in PMI, the risk contribution of the ε4 allele may be diluted by the data of the general population due to PMI patients have a higher genetic load. Secondly, ethnic differences significantly affect the research conclusions. In the Chinese population, the frequency of the ε4 allele shows a north-south gradient distribution [31]. The existing studies have insufficient targeted analysis of PMI in different populations, making it difficult to directly guide clinical practice. Thirdly, there are differences in the research conclusions regarding the risk effect of *APOE* genotypes and MI [10, 32].

This study focuses on the association between *APOE* genotypes and PMI. Through a hospital-based retrospective case-control design, it systematically analyzed the differences in genotype distributions between PMI patients and healthy controls, while adjusting for confounding factors such as lipid levels and lifestyle. The research results are expected to clarify the risk effect intensity of *APOE* genotypes in PMI among the Han population in South China, providing a basis for constructing an early risk prediction model based on genetic marker plus clinical indicator.

## Materials and methods

### Study participants and data collection

As a retrospective study, this research consecutively enrolled 1007 subjects who were admitted to Meizhou People’s Hospital from December 2018 to March 2024. Among them, there were 379 PMI patients and 628 age-matched individuals without AMI (controls). This study was supported by the Ethics Committee of the Meizhou People’s Hospital.

For the diagnosis of acute myocardial infarction (AMI), two key conditions must be met concurrently. First, markers indicating acute myocardial injury should show elevations or reductions, with at least one measurement exceeding the 99th percentile of the upper reference limit. Second, the patient must present with at least one of the following clinical manifestations: (1) symptoms characteristic of acute myocardial ischemia; (2) newly developed ischemic changes on electrocardiogram; (3) emergence of new pathological Q-waves; (4) imaging findings that confirm recent loss of viable myocardial tissue or abnormal segmental motion of the ventricular wall; and (5) verification of coronary thrombosis via coronary angiography or intracavitary imaging techniques.

Inclusion criteria of patients: (1) patients diagnosed with AMI; (2) the age of onset met the criteria for PMI (for males, ≤ 55 years old; for females, ≤ 65 years old); (3) the clinical data were complete (including medical history, physical examination, laboratory tests, and imaging data); (4) they voluntarily participated in this study. Exclusion criteria: (1) secondary myocardial infarction (such as myocardial injury caused by myocarditis, cardiomyopathy, pulmonary embolism, and aortic dissection); (2) complicated with malignant tumors, severe liver or kidney dysfunction, autoimmune diseases or hereditary metabolic disorders; (3) had coronary artery bypass surgery or percutaneous coronary intervention in the past; (4) had a major surgical history or severe infection within the last 3 months; (5) clinical data is incomplete.

Inclusion criteria for the control group: (1) had undergone health check-ups at the hospital’s health center during the same period, and no evidence of cardiovascular diseases was found through physical examination, electrocardiogram, cardiac ultrasound, and lipid testing; (2) age was matched with that of the case group; (3) had no history of cardiovascular diseases such as myocardial infarction, coronary heart disease, or stroke; and (4) volunteered to participate in this study.

### Data collection and classification of indicators

Through the hospital’s electronic medical record system, the basic information of the research subjects (gender, age, smoking history, drinking history, history of hypertension, history of diabetes) and laboratory test results (triglycerides, total cholesterol, low-density lipoprotein cholesterol, high-density lipoprotein cholesterol) were retrospectively collected.

Hypertension is diagnosed when the mean systolic blood pressure (SBP) exceeds 140 mmHg and/or the mean diastolic blood pressure (DBP) is over 90 mmHg [33]. Dyslipidemia may be diagnosed if any one of the following conditions is fulfilled, as specified in the Guidelines for the Prevention and Control of Dyslipidemia in Chinese Adults: (1) total cholesterol (TC) ≥ 6.22 mmo1/L), (2) triglyceride (TG) ≥ 2.26 mmo1/L, or (3) low-density lipoprotein-cholesterol (LDL-C) ≥ 4.14 mmo1/L [34, 35]. Data including age, gender, smoking history, alcohol consumption history, diabetes mellitus history, and hypertension history were retrieved from the patients’ electronic medical record system.

### APOE genotyping

Genomic DNA was isolated from venous blood samples that had been collected in EDTA anticoagulant tubes, with the use of a blood DNA extraction kit supplied by Qiagen GmbH (Germany). A Nano-Drop 2000™ spectrophotometer (ThermoFisher Scientific, USA) was employed to evaluate both the quality and concentration of the extracted DNA. Subsequently, polymerase chain reaction (PCR) was employed to amplify *APOE* gene polymorphisms under the following thermal cycling conditions: an initial pre-denaturation step at 50℃ for 2 min and 95℃ for 15 min, followed by 45 cycles of denaturation at 94℃ for 30 s and annealing at 65℃ for 45 s. All PCR reagents utilized in this procedure were supplied by Sinochips Bioscience Co., Ltd. (China)(Registration Certificate Number of the National Medical Products Administration of China: National Medical Device Approval 20183400388). After amplification, the resultant PCR products were subjected to hybridization with wild-type and mutant probes immobilized on the gene chip, and sample genotypes were further identified based on the hybridization signals generated. To ensure the accuracy and validity of experimental outcomes, positive controls, negative controls, and blank controls were incorporated throughout the testing process. Only when all three types of controls yielded consistent and expected results were the batch test data deemed reliable.

### Statistical analysis

Continuous variables were presented as means ± standard deviations (SD), with group comparisons conducted using either Student’s t-test or the Mann-Whitney U test, depending on data distribution. Hardy-Weinberg equilibrium among the study participants was assessed, and comparisons of genotype distribution ratios and allele frequencies between the two groups were analyzed via the χ^2^ test. To explore the associations between the genotypes and alleles of *APOE* and PMI, univariate analysis and multivariate logistic regression analysis were employed. A *p* value of less than 0.05 was regarded as statistically significant. All statistical analyses were carried out using SPSS Statistics software (Version 26.0, IBM Inc., USA).

## Results

### Characteristics of subjects

Among the subjects included in this study, 658(65.3%) were men and 349(34.7%) were women; median age was 51.0 (IQR: 45.0, 54.0) years. There were 276(27.4%), 62(6.2%), 308(30.6%), 405(40.2%), and 345(34.3%) subjects had history of smoking, history of alcohol consumption, hypertension, diabetes mellitus, and dyslipidemia, respectively. The proportions of history of smoking (38.3% vs. 20.9%, *p <* 0.001), hypertension (49.9% vs. 18.9%, *p <* 0.001), and dyslipidemia (44.6% vs. 28.0%, *p <* 0.001) in PMI patients were higher than those in controls. The patients with PMI had higher TC (5.24 (4.35, 6.04) vs. 4.69 (3.92, 5.51) mmol/L, *p* < 0.001) and TG (1.83 (1.33, 2.66) vs. 1.32 (0.92, 2.09) mmol/L, *p* < 0.001), and lower HDL-C (2.54 (2.06, 3.16) vs. 2.91 (2.35, 3.55) mmol/L, *p* < 0.001) levels than controls. There was no significant difference in age, gender distribution, history of alcohol consumption and diabetes mellitus between PMI patients and controls (all *p* > 0.05) (Table 1).

**Table 1:** Clinical characteristics of subjects in this study

Variables	Total (*n* = 1007)	Controls (*n* = 628)	PMI patients (*n* = 379)	*p* values
Age (years), median (IQR)	51.0 (45.0, 54.0)	50.0 (43.0, 54.0)	52.0 (48.0, 55.0)	0.081 (Z=−1.743)
Gender				
Male, n(%)	658(65.3%)	396(63.1%)	262(69.1%)	0.056 (χ^2^=3.848)
Female, n(%)	349(34.7%)	232(36.9%)	117(30.9%)	
History of smoking				
No, n(%)	731(72.6%)	497(79.1%)	234(61.9%)	< 0.001 (χ^2^=35.961)
Yes, n(%)	276(27.4%)	131(20.9%)	145(38.3%)	
History of alcohol consumption				
No, n(%)	945(93.8%)	588(93.6%)	357(94.2%)	0.787 (χ^2^=0.130)
Yes, n(%)	62(6.2%)	40(6.4%)	22(5.8%)	
Hypertension				
No, n(%)	699(69.4%)	509(81.1%)	190(50.1%)	< 0.001 (χ^2^=106.427)
Yes, n(%)	308(30.6%)	119(18.9%)	189(49.9%)	
Diabetes mellitus				
No, n(%)	602(59.8%)	380(60.5%)	222(58.6%)	0.551 (χ^2^=0.368)
Yes, n(%)	405(40.2%)	248(39.5%)	157(41.4%)	
Levels of serum lipid				
TC, mmol/L, median (IQR)	4.85 (4.06, 5.76)	4.69 (3.92, 5.51)	5.24 (4.35, 6.04)	< 0.001 (Z=−6.002)
TG, mmol/L, median (IQR)	1.50 (1.04, 2.27)	1.32 (0.92, 2.09)	1.83 (1.33, 2.66)	< 0.001 (Z=−8.075)
HDL-C, mmol/L, median (IQR)	1.15 (0.97, 1.38)	1.19 (0.98, 1.42)	1.11 (0.96, 1.31)	< 0.001 (Z=−3.683)
LDL-C, mmol/L, median (IQR)	2.68 (2.13, 3.27)	2.91 (2.35, 3.55)	2.54 (2.06, 3.16)	< 0.001 (Z=−5.737)
Dyslipidemia				
No, n(%)	662(65.7%)	452(72.0%)	210(55.4%)	< 0.001 (χ^2^=28.798)
Yes, n(%)	345(34.3%)	176(28.0%)	169(44.6%)	

*TC* total cholesterol, *TG* triglycerides, *HDL-C* high-density lipoprotein-cholesterol, *LDL-C* low-density lipoprotein-cholesterol

### Distribution frequencies of *APOE* genotypes and alleles in patients with PMI and controls

There were 15(1.5%), 147(14.6%), 16(1.6%), 670(66.5%), 149(14.8%), and 10(1.0%) individuals with *APOE* ɛ2/ɛ2, ɛ2/ɛ3, ɛ2/ɛ4, ɛ3/ɛ3, ɛ3/ɛ4, and ɛ4/ɛ4 genotype, respectively. The *APOE* genotype distribution in all subjects was consistent with Hardy-Weinberg equilibrium (*χ*^2^ = 4.679, *p* = 0.322). Similarly, the genotype distribution conformed to Hardy-Weinberg equilibrium in both the PMI patient group (*χ*^2^ = 0.879, *p* = 0.928) and controls (*χ*^2^ = 3.972, *p* = 0.410).

Compared with the control group, the frequency of the *APOE* ɛ2/ɛ3 genotype in PMI patients was significantly lower (7.7% vs. 18.8%, *p* < 0.001), while that of ɛ3/ɛ4 genotype was notably higher (20.6% vs. 11.3%, *p* < 0.001). For alleles, the frequency of ε2 was lower in PMI patients than in controls (4.4% vs. 12.7%, *p* < 0.001), whereas the frequency of ε4 was higher (12.1% vs. 7.4%, *p* < 0.001) (Table 2).

**Table 2:** Distribution frequencies of *APOE* genotypes and alleles in patients with PMI and controls

Variable	Genotype/allele	Total (*n* = 1007)	Controls (*n* = 628)	PMI patients (*n* = 379)	χ^2^	*p* values
*APOE* genotype						
	ɛ2/ɛ2	15(1.5%)	15(2.4%)	0(0)	9.189	0.002
	ɛ2/ɛ3	147(14.6%)	118(18.8%)	29(7.7%)	23.520	< 0.001
	ɛ2/ɛ4	16(1.6%)	12(1.9%)	4(1.1%)	1.106	0.317
	ɛ3/ɛ3	670(66.5%)	407(64.8%)	263(69.4%)	2.231	0.148
	ɛ3/ɛ4	149(14.8%)	71(11.3%)	78(20.6%)	16.127	< 0.001
	ɛ4/ɛ4	10(1.0%)	5(0.8%)	5(1.3%)	0.658	0.515
HWE (χ^2^, *p*)		χ^2^ = 4.679, *p* = 0.322	χ^2^ = 3.972, *p* = 0.410	χ^2^ = 0.879, *p* = 0.928		
*APOE* allele						
	ε2	193(9.6%)	160(12.7%)	33(4.4%)	38.361	< 0.001
	ε3	1636(81.2%)	1003(79.9%)	633(83.5%)	4.137	0.045
	ε4	185(9.2%)	93(7.4%)	92(12.1%)	12.693	< 0.001

*HWE* Hardy Weinberg Equilibrium

### Clinical characteristics of subjects stratified by *APOE* ɛ2, ɛ3, ɛ4 alleles

Due to the opposite roles of ɛ2 and ɛ4 alleles in lipid metabolism, individuals with the ɛ2/ɛ4 genotype (*n* = 16) were excluded from the analysis of the correlation between *APOE* alleles and patients’ clinical characteristics. Significant differences were observed between PMI patients and controls in gender distribution (*p* < 0.001) and smoking history proportion (*p* = 0.002). However, no significant differences were found in age, the proportions of alcohol consumption history, hypertension, or diabetes mellitus between the two groups (all *p* > 0.05). Additionally, the TC level in patients carrying the *APOE* ɛ2 allele (4.89 (4.24, 5.84) mmol/L) was significantly higher than that in those with ɛ3 (4.89 (4.07, 5.86) mmol/L) and ɛ4 alleles (4.60 (3.81, 5.39) mmol/L), respectively (*p* = 0.007) (Table 3).

**Table 3:** Clinical characteristics of subjects stratified by *APOE* ɛ2, ɛ3, ɛ4 alleles

Variables	ε2 (ɛ2/ɛ2 + ɛ2/ɛ3) (*n* = 162)	ε3 (ɛ3/ɛ3) (*n* = 670)	ε4 (ɛ3/ɛ4 + ɛ4/ɛ4) (*n* = 159)	*p* values
Age (years), median (IQR)	52.0 (46.8, 55.0)	50.5 (45.0, 54.0)	51.0 (46.0, 55.0)	0.197
Gender				
Male, n(%)	89(54.9%)	424(63.3%)	131(82.4%)	< 0.001 (χ^2^=29.206)
Female, n(%)	73(45.1%)	246(36.7%)	28(17.6%)	
History of smoking				
No, n(%)	127(78.4%)	494(73.7%)	98(61.6%)	0.002 (χ^2^=12.761)
Yes, n(%)	35(21.6%)	176(26.3%)	61(38.4%)	
History of alcohol consumption				
No, n(%)	155(95.7%)	627(93.6%)	147(92.5%)	0.450 (χ^2^=1.516)
Yes, n(%)	7(4.3%)	43(6.4%)	12(7.5%)	
Hypertension				
No, n(%)	110(67.9%)	470(70.1%)	107(67.3%)	0.717 (χ^2^=0.676)
Yes, n(%)	52(32.1%)	200(29.9%)	52(32.7%)	
Diabetes mellitus				
No, n(%)	103(63.6%)	387(57.8%)	98(61.6%)	0.325 (χ^2^=2.246)
Yes, n(%)	59(36.4%)	283(42.2%)	61(38.4%)	
Levels of serum lipid				
TC, mmol/L, median (IQR)	4.60 (3.81, 5.39)*^,$^	4.89 (4.07, 5.86)^#^	4.89 (4.24, 5.84)^#^	0.007
TG, mmol/L, median (IQR)	1.51 (0.98, 2.28)	1.48 (1.03, 2.25)	1.62 (1.12, 2.53)	0.224
HDL-C, mmol/L, median (IQR)	1.20 (0.98, 1.42)	1.15 (0.97, 1.37)	1.12 (0.96, 1.32)	0.125
LDL-C, mmol/L, median (IQR)	2.35 (1.87, 2.84)*^,$^	2.76 (2.17, 3.33)^#^	2.77 (2.33, 3.51)^#^	0.001
Dyslipidemia				
No, n(%)	110(67.9%)	440(65.7%)	100(62.9%)	0.646 (χ^2^=0.898)
Yes, n(%)	52(32.1%)	230(34.3%)	59(37.1%)	

*TC* total cholesterol, *TG* triglycerides, *HDL-C* high-density lipoprotein-cholesterol, *LDL-C* low-density lipoprotein-cholesterol

^#^compared with ε2, *p* < 0.05;

*compared with ε3, *p* < 0.05;

^$^compared with ε4, *p* < 0.05

### Logistic regression analysis of risk factors of PMI

In univariate analysis, history of smoking (odds ratio (OR): 2.351, 95% confidence interval (CI): 1.772–3.119, *p* < 0.001), hypertension (OR: 4.255, 95% CI: 3.204–5.649, *p* < 0.001), dyslipidemia (OR: 2.067, 95% CI: 1.582-2.700, *p* < 0.001), *APOE* ɛ3/ɛ4 genotype (ɛ3/ɛ4 vs. ɛ3/ɛ3, OR: 1.700, 95% CI: 1.190–2.429, *p* = 0.004), and *APOE* ɛ4 allele (ɛ4 vs. ɛ3, OR: 1.690, 95% CI: 1.194–2.393, *p* = 0.003) were significantly associated with PMI.

Multivariate logistic regression revealed the following independent associated factors for PMI: history of smoking (OR: 3.057, 95% CI: 2.098–4.455, *p* < 0.001), hypertension (OR: 4.474, 95% CI: 3.273–6.117, *p* < 0.001), dyslipidemia (OR: 1.805, 95% CI: 1.333–2.443, *p* < 0.001), and *APOE* ɛ3/ɛ4 genotype (ɛ3/ɛ4 vs. ɛ3/ɛ3, OR: 1.548, 95% CI: 1.038–2.309, *p* = 0.032), and *APOE* ɛ4 allele (ɛ4 vs. ɛ3, OR: 1.521, 95% CI: 1.033–2.241, *p* = 0.034) (Table 4).

**Table 4:** Logistic regression analysis of risk factors of PMI

Variables	Univariate OR (95% CI)	*p* values	Multivariate OR (95% CI)	*p* values
Age	1.057 (1.041–1.075)	0.078	1.029 (1.005–1.052)	0.061
Gender (male/female)	1.312 (1.000-1.721)	0.050	1.066 (0.730–1.557)	0.074
History of smoking (yes/no)	2.351 (1.772–3.119)	< 0.001	3.057 (2.098–4.455)	< 0.001
History of alcoholism (yes/no)	0.906 (0.530–1.549)	0.718	0.458 (0.243–0.865)	0.016
Hypertension (yes/no)	4.255 (3.204–5.649)	< 0.001	4.474 (3.273–6.117)	< 0.001
Diabetes mellitus (yes/no)	1.084 (0.836–1.405)	0.544	0.776 (0.575–1.049)	0.099
Dyslipidemia (yes/no)	2.067 (1.582-2.700)	< 0.001	1.805 (1.333–2.443)	< 0.001
*APOE* genotypes				
ɛ3/ɛ3	1.000 (reference)	-	1.000 (reference)	-
ɛ2/ɛ2	-	0.998	-	0.998
ɛ2/ɛ3	0.380 (0.246–0.587)	< 0.001	0.309 (0.191-0.500)	< 0.001
ɛ2/ɛ4	0.516 (0.165–1.616)	0.256	0.522 (0.153–1.785)	0.300
ɛ3/ɛ4	1.700 (1.190–2.429)	0.004	1.548 (1.038–2.309)	0.032
ɛ4/ɛ4	1.548 (0.444–5.397)	0.493	1.106 (0.286–4.276)	0.884
*APOE* allele				
ε3	1.000 (reference)	-	1.000 (reference)	-
ε2	0.337 (0.219–0.519)	< 0.001	0.264 (0.164–0.423)	< 0.001
ε4	1.690 (1.194–2.393)	0.003	1.521 (1.033–2.241)	0.034

*OR* odds ratio, *CI *confidence interval

## Discussion

In this study, history of smoking, hypertension, dyslipidemia, and *APOE* ɛ3/ɛ4 genotype were independently associated with PMI. It indicates that individuals with a history of smoking, hypertension, dyslipidemia, and carrying the *APOE* ε3/ε4 genotype should be vigilant against PMI. The *APOE* ε3/ε4 genotype was found to be significantly associated with PMI, and thus it may serve as a potential genetic marker for PMI.

Some studies found that *APOE* ε4 allele may be associated with CAD risk in Finnish adults [36], Egyptians [37], ethnic Kashmiri population [38], and Chinese populations [39, 40]. However, some studies have found that *APOE* polymorphisms were not associated with the susceptibility of coronary artery atherosclerosis (CAD) [41, 42]. There have also been some studies on the relationship between *APOE* polymorphisms and PMI. *APOE* ε4 allele was associated with PMI in a Germany population [43], in Asian Indians [44]. The research conducted by Brscic E et al.. indicated that the *APOE* polymorphism was also associated with the prognosis of acute myocardial infarction in young adults [45]. However, several studies have indicated that *APOE* polymorphism is not associated with early-onset cardiovascular diseases. For instance, a study conducted on the Iraqi population demonstrated no significant correlation between *APOE* polymorphism and PMI [10]; another study carried out on a Italian population showed that *APOE* polymorphism does not correlate with the incidence of early-onset occurrence of acute coronary syndrome [32].

From a biological mechanism perspective, the risk effect of the ε3/4 genotype may stem from the alteration of the function of the ApoE protein. The ApoE protein encoded by the ε4 allele has a lower affinity for the low-density lipoprotein receptor (LDLR) compared to the ε3 allele, resulting in a decrease in lipid metabolism efficiency, delayed clearance of plasma LDL-C, and thereby accelerating the formation of atherosclerotic plaques [46, 47]. More importantly, the ε3/4 genotype not only promotes lipid deposition by increasing LDL-C, but also activates the inflammatory pathways within the vascular wall. Studies have confirmed that the serum C-reactive protein (CRP) and tumor necrosis factor-α (TNF-α) levels of ε4 allele carriers are significantly higher than those of ε3/3 genotype carriers, and the inflammatory response further disrupts the stability of the plaque fibrous cap and increases the risk of acute thrombosis [48, 49]. In this study, the smoking rate of ε4 allele carriers was significantly higher than that of the control group (*p* = 0.002), suggesting that this environmental factor, smoking, may have a synergistic effect with the ε4 allele, exacerbating oxidative stress and vascular endothelial damage, and jointly increasing the risk of PMI, which is consistent with the theory that genetic-environmental interactions are involved in the cardiovascular diseases [50, 51].

PMI is associated with traditional cardiovascular risk factors such as smoking, diabetes mellitus, and hypertension [2, 52–55], and there are differences in gender [53]. Most studies have largely identified smoking as a risk factor for PMI [56–58]. In this study, history of smoking and hypertension were independent risk factors for PMI. In addition, the vast majority of studies revealed that dyslipidemia was associated with PMI risk [54, 55, 57, 59, 60]. This study has found that dyslipidemia (OR: 1.811, *p* < 0.001), was also a risk factor for PMI. The results of this study are consistent with those of the majority of previous studies.

### Study strength and limitations

The results of this study provide an important basis for precise risk stratification and intervention of PMI. For high-risk individuals of PMI, *APOE* genotype testing can be included in the screening program. If the test result is ε3/ε4 genotype, clinical monitoring should also be strengthened. At the same time, targeted lifestyle interventions (such as quitting smoking and following a low-fat diet) can be carried out. If necessary, heparin-based primary prevention drugs can be initiated in advance to reduce the risk of PMI. However, this study has some limitations. Firstly, this study is a single-center retrospective design. The case group is solely composed of inpatients from the cardiovascular department of our hospital, while the control group consists of the general population undergoing physical examinations. This may not fully represent the general population and there may be selection bias. Future multi-center prospective studies are needed to verify the results. Secondly, the expression level of ApoE protein and the polymorphisms of other related genes (such as LDLR and PCSK9) were not detected. This prevented in-depth exploration of the interaction between genes and proteins as well as between genes and proteins on the impact of PMI. Thirdly, this study lacks information regarding the medication history of the enrolled patients. Detailed data on the medications used by the subjects both before and after disease onset were not collected. Consequently, we cannot rule out the confounding effect of pharmacotherapy on the association analysis between *APOE* genotypes and PMI risk, nor can we clarify whether pharmacotherapy modifies the impact of genotypes on the incidence risk of PMI. Finally, long-term prognosis data (such as the recurrence rate of myocardial infarction and mortality rate) of the study subjects were not collected. Therefore, it is impossible to determine whether the ε3/ε4 genotype is related to the prognosis of PMI patients. Further studies can expand the follow-up analysis.

Based on the results and limitations of this study, the following directions are suggested for future research. First, conduct multi-center, large-sample prospective cohort studies to further verify the association between the *APOE* ɛ3/ɛ4 genotype and PMI, and extend the follow-up period to observe its impact on disease prognosis (such as recurrent myocardial infarction and mortality risk). Second, combine genome-wide association analysis (GWAS) or polygenic risk score (PRS) to explore the combined effect of the *APOE* genotype with other genetic loci, and construct a more accurate genetic risk prediction model for PMI. Third, conduct mechanistic studies to clarify the specific molecular pathways through which the *APOE* ɛ3/ɛ4 genotype affects PMI (such as whether it affects lipid metabolism, vascular endothelial function or inflammatory response). Finally, verify whether intensified management (such as targeted lipid-lowering drugs, lifestyle intervention) can reduce the risk of PMI, and provide higher-level evidence for clinical practice.

## Conclusion

Based on a retrospective analysis of a hospital-based cohort, this study investigated the correlation between *APOE* polymorphisms and PMI. The results demonstrated that the *APOE* ε3/ε4 genotype was significantly associated with PMI, suggesting that this genotype could serve as a potential genetic marker for PMI risk assessment. This finding provides a novel genetic perspective for the screening and formulation of personalized preventive intervention strategies for PMI. However, the rersults of this study need to be further verified by prospective studies, and the underlying mechanisms should be clarified through basic experimental research, so as to provide more robust scientific evidence for the precise prevention and control of PMI.

## Data Availability

The datasets used and analyzed during the current study available from the corresponding author on request.
